# CESR development programme at LSCFT

**DOI:** 10.1192/bjb.2022.70

**Published:** 2022-12

**Authors:** Qutub Jamali, Muhammad Iqbal Naeem

**Affiliations:** Specialty Doctor, Old Age Psychiatry, Lancashire & South Cumbria NHS Foundation Trust, UK. Email: qutub.jamali@lscft.nhs.uk; Consultant Psychiatrist, Lancashire & South Cumbria NHS Foundation Trust, UK

04 July 2022

In 2019, Lancashire & South Cumbria NHS Foundation Trust (LSCFT) started the CESR (Certificate of Eligibility for Specialist Registration)^1^ development programme in old age psychiatry^2^ to support applicants who wish to progress towards CESR and potentially substantive consultant positions. It is a 3 year rotation programme, which includes yearly rotation in in-patient, community and liaison settings. This post is designed to support applicants in broadening their skills in old age psychiatry and developing necessary competencies to successfully achieve CESR. This leading step in LSCFT involved setting up a CESR Peer Support Group for candidates (for mutual learning from each others’ experience with support from a senior consultant with experience of CESR evaluation and coaching). Since May 2021, the Trust has taken a further step to set up a programme for mentorship and coaching to provide robust support and guidance for CESR doctors, which is being received very well by the candidates. The support provided by the Trust includes:

- two sessions supporting professional activities time as standard;
- guidance to compile evidence by weekly supervision from clinical supervisor;
- monthly meeting with CESR mentor for guidance regarding CESR progression;
- monthly mandatory attendance in CESR peer group for further advice and guidance on CESR evidence, which includes discussion on intended learning outcomes in detail;
- free access to RCPsych's CPD Online;
- opportunities for involvement in research and allocation of research supervisor; support from dedicated research and development team in the Trust;
- opportunities to develop leadership and management opportunities by leading or chairing quality improvement projects;
- opportunities to be involved in teaching at various levels, including an opportunity to work as a medical education fellow;
- medical education fellowship opportunity to develop understanding of various dimensions of medical education and develop skills in organising various educational events in the Trust;
- special interest sessions for CESR: as this is a CESR development post in old age psychiatry, support is provided for special interest sessions in general adult and child and adolescent mental health services;
- special interest sessions in geriatric medicine and appraiser training provided;
- annual review of competency progression conducted to monitor CESR progression;
- specialty doctors who are not in this post and acting consultants working in the Trust are also offered the opportunity to work on their CESR portfolio.

This programme was started in 2019 and has proved to be significantly beneficial to the specialty doctors in the Trust. One of the specialty doctors who pursued this programme stated that the ‘CESR development programme at LSCFT was an excellent opportunity to develop the skills of a competent clinician, teacher, appraiser, researcher and manager without compromising on work–life balance. This was only possible due to the nature of evidence required for CESR, i.e. primary evidence which includes clinical case histories, creating teaching opportunities, preparing teaching presentations, developing research protocols, experience of research ethical approval and quality improvement projects’.

Although the CESR journey can be a daunting task, if appropriate support is provided by the Trust, it is certainly achievable.
